# Cancer therapeutic targeting using mutant–p53-specific siRNAs

**DOI:** 10.1038/s41388-018-0652-y

**Published:** 2019-01-14

**Authors:** Ifeoma Ubby, Christian Krueger, Roberto Rosato, Wei Qian, Jenny Chang, Kanaga Sabapathy

**Affiliations:** 1 Division of Cellular & Molecular Research, Humphrey Oei Institute of Cancer Research, National Cancer Centre Singapore, Singapore, 169610 Singapore; 2 Houston Methodist Cancer Center, 6445 Main St, Houston, TX 77030 USA; 3 Cancer and Stem Cell Biology Program, Duke-NUS Medical School, Singapore, 169857 Singapore; 4 Department of Biochemistry, Yong Loo Lin School of Medicine, National University of Singapore, Singapore, 119228 Singapore; 5 Institute of Molecular & Cellular Biology, Singapore, 138673 Singapore

**Keywords:** Biochemistry, Cancer

## Abstract

Mutations in *Tp53* compromise therapeutic response, due either to the dominant-negative effect over the functional wild-type allele; or as a result of the survival advantage conferred by mutant p53 to which cancer cells become addicted. Thus, targeting mutant p53 represents an effective therapeutic strategy to treat over half of all cancers. We have therefore generated a series of small-interfering-RNAs, capable of targeting four p53 hot-spot mutants which represent ~20% of all p53 mutations. These mutant–p53-specific siRNAs (MupSi) are highly specific in silencing the expression of the intended mutants without affecting wild-type p53. Functionally, these MupSis induce cell death by abrogating both the addiction to mutant p53 and the dominant-negative effect; and retard tumor growth in xenografts when administered in a therapeutic setting. These data together demonstrate the possibility of targeting mutant p53 specifically to improve clinical outcome.

## Introduction

A decade of pan-cancer genome sequencing efforts have resulted in the identification of a large number of genomic alterations across almost all cancer types [1, 2]. This has led to the characterization and association of many of these mutations as potential drivers that are casually involved in cancer development. Some of the identified alterations in oncogenes have been subjected to therapeutic targeting through the development of inhibitory molecules or blocking antibodies, which have had huge initial success in the treatment of cancers bearing these alterations [3–5], forming the basis of precision medicine in Oncology. A case in point is the V600E activating mutation that has been identified in B-Raf in many cancers including melanomas; and vemurafenib, an inhibitor that is effective in controlling the growth of tumors with these V600E mutations [6]. However, one challenge of such an approach of using inhibitors or blocking antibodies for therapeutic targeting is that they are not entirely specific for the mutant form of the protein, but rather, are more effective in mutant protein expressing cells due to the elevated activity or expression of the mutant protein over its wild-type (WT) counterpart, and also affects other related targets [7, 8]. This therefore has the potential to lead to undesirable side effects on other cell-types that express the WT protein [9, 10].

An ideal drug against a mutated protein would therefore be one that will only affect the functioning of the mutant form, without any effects on the WT version. However, up till now and to our knowledge, no drugs or molecules have been generated that are capable of such high specificity. A very recent report has identified a much more potent inhibitor of mutant c-Kit, with dramatically reduced effects on the WT proteins [7]. Nonetheless, no routine technology to generate “mutant-only”-specific reagents is yet available.

Among the mutated genes in cancers, mutations in the tumor suppressor gene *Tp53* (hereinafter referred to as *p53*) occur with the highest frequency [1, 2, 11], cementing its position as the critical gate-keeper gene whose functions have to be abrogated for cancers to develop [12, 13]. Mutations in *p53* can occur almost on all of its 393 residues [2], and these mutations impact tumorigenesis in multiple ways. Firstly, mutations in *p53* in the germ line lead to cancer predisposition, as exemplified in the Li−Fraumeni syndrome, and in many model organisms [14–18]. In addition, mutations in *p53* have been associated with poor response to therapy, due often to the dominant-negative (DN) effects of the mutant protein over the remaining WT protein, which could be ameliorated by reducing the expression of the mutant form [19–25]. Finally, cancer cells are often addicted to the presence of mutant p53 for survival and metastasis, and abrogation of many of the acquired gain-of-functions (GOF) of mutant p53 can reduce addiction and metastasis, thereby inducing tumor cell death and tumor load in vivo [26–32]. However, GOF in itself appears not to be a universal phenomenon among all p53 mutants [22, 32].

From the therapeutic perspective, mutant p53 would therefore be expected to be the prime target to treat cancers. However, the abysmal lack of enthusiasm to develop reagents to target mutant p53 stems from its persona as an “undruggable” transcription factor, which has hampered progress for decades [23]. Moreover, recent emerging data indicate that not all mutants are equal in form and function, and hence, targeting mutant p53 would require a plethora of molecules (rather than a single agent) capable of selectively targeting the various p53 mutants, all of which should not affect the functioning of the WT form to be effective [23]. Thus, current technologies used in drug development have not been applied or been successful in targeting mutant p53 yet.

Small-interfering RNAs (siRNA) have been developed for many targets to silence their expression successfully [33, 34], and can be seen as a potential tool to target the various p53 mutants. However, siRNAs that are capable of recognizing only a single nucleotide change have not been generated routinely, due mainly to the inability to achieve specificity to target a single nucleotide change, without affecting the WT counterparts of the intended targets. Nonetheless, occasional reports have demonstrated the use of siRNAs selective for a single nucleotide change [35–39], and proof-of-concept has been shown with an siRNA against the R248W p53 mutant [35]. These technologies have not been utilized routinely to generate reagents for multiple genetic alterations on the same gene. We have therefore explored the possibility of expanding on this technology to generate siRNAs that are specific for four of the six hot-spot mutations of p53: R175H, R248W, R249S, and R273H, which account for about 20% of all p53 mutations noted [2, 11]. In this report, we demonstrate the generation of such mutant p53-specific siRNAs (referred to as MupSi), and demonstrate their ability in selectively silencing the expression of the intended mutant p53 forms, without cross-reactivity against other mutants or against the WT protein. Furthermore, these siRNAs have been used to demonstrate the amelioration of the DN activity of mutant p53 over the WT form, thereby sensitizing tumor cells to therapeutic treatment. Moreover, they also abrogate the addiction of cancer cells to mutant p53 for survival, leading to cell death of tumor cells expressing mutant p53. Finally, we show that these siRNAs can be used as therapeutic agents in patient-derived xenografts, and are capable of retarding tumor growth in vivo without resulting in any side effects or organ toxicity. Together, these data demonstrate that mutation-specific siRNAs can be routinely generated, and this approach could therefore be expanded to target the other major tumor suppressors and oncogenes that are altered in various pathological conditions.

## Results

### Design and selection of allele-specific siRNAs for hot-spot p53 mutants

**Fig. 1:**
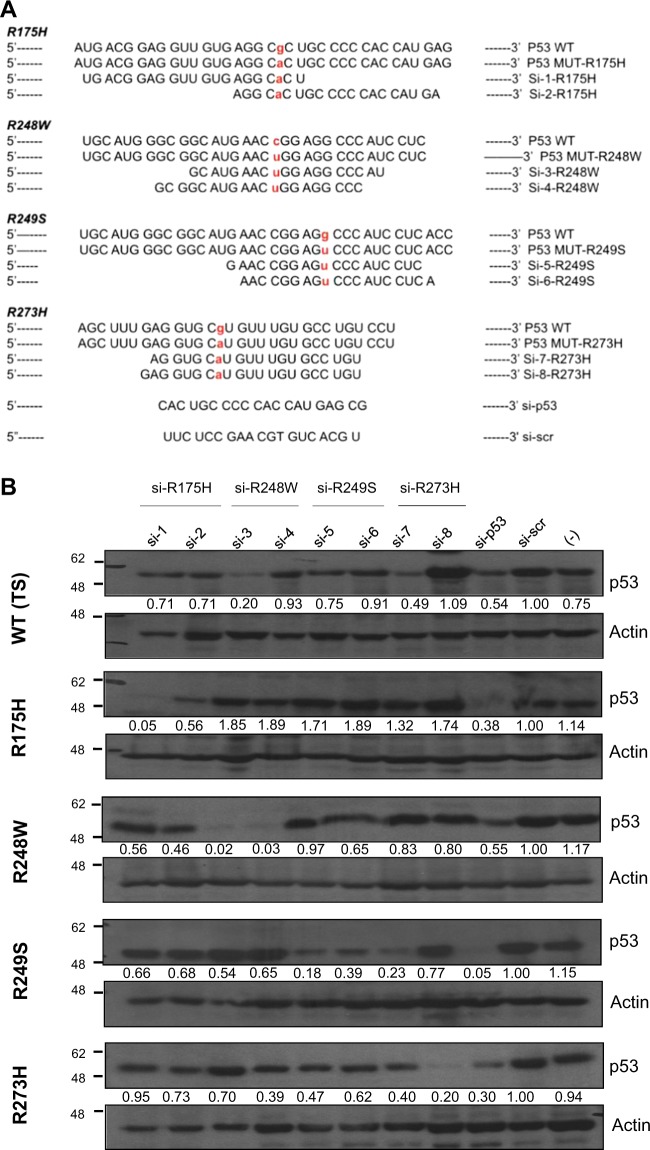
siRNA sequences selected by siRNA walk to specifically target various p53 hot-spot mutants. **a**, **b.** Nucleotide sequence of wild-type (WT) and the respective p53 mutants (i.e. R175H, R248W, R249S, and R273H) are indicated in each case, followed by the p53 allele-specific siRNA sequences shortlisted to target each mutant. Both the WT and the mutated nucleotide residue are highlighted in bold and red. The pan-p53 siRNA (si-p53) and the scrambled (scr) siRNA (si-scr) sequences are indicated at the bottom (**a**). Each siRNA was transfected into isogenic H1299 cell lines stably expressing the indicated p53 mutants, and cell lysate was analyzed for p53 expression by immunoblotting, 72 h post-transfection using anti-p53 antibody (DO-1). Temperature-sensitive (TS) WT p53-expressing cells were used as a WT control. One representative blot of at least three independent experiments is shown. Actin is shown as loading control. (−) represent cells only without any siRNA transfection (**b**). For each sample, the ratio of p53 to Actin band intensity was calculated and normalized to the ratio of si-scr control. Values represent normalized fold change

We embarked on generating siRNAs that will be capable of only silencing the mutant *p53* alleles, without having an impact on WT p53 expression. To this end, we generated a library of a large number of siRNAs, by performing sequence walks such that the position of the mutant nucleotide was varied with respect to the entire siRNA strand. All the siRNAs were transfected in a series of H1299-based isogenic cell lines which stably expressed the various p53 mutants, or the temperature-sensitive (TS) WT p53 [27], and data from representative siRNAs that show specific activity against the four hot-spot mutants: R175H, R248W, R249S, and R273H are shown (Fig. 1a). These were among the few shortlisted siRNAs that had specific activity for the respective p53 mutants. All the indicated siRNAs were transiently transfected in all isogenic cell lines, which were harvested for p53 protein expression analysis 72 h later, by immunoblotting. As shown in Fig. 1b, si-p53, which targets all p53 indiscriminately, was capable of reducing the expression of p53 in all cell lines, compared to scrambled siRNA or cells that were not transfected (last three lanes on the right of the gels). Most of the mutation-specific siRNAs showed specificity and were able to discriminate the intended mutants, with reduced to negligible effects on other mutants or the WT p53: for instance, si-1 and si-2, which are specific for R175H mutant p53, were capable of reducing R175H expression, but had minimal impact on the other p53 mutants and WT p53 in this cell system. Similarly, si-4 which is specific for R248W mutant p53 was capable of markedly reducing the expression of R248W mutant, without impacting other mutants. On the other hand, however, si-3 which also targets the R248W mutant, though capable of reducing the expression of its intended mutation, also led to a decrease in the expression of WT p53. Similarly, while si-8 which targets R273H was very specific, si-7 also had some effects on both WT p53 and the R249S mutant. Collectively, these data indicate that evaluation of multiple siRNAs generated against the same mutation on multiple cell systems is crucial to obtain highly mutant-specific reagents.

### Mutation-specific siRNA-mediated silencing of endogenous mutant p53 expression

**Fig. 2:**
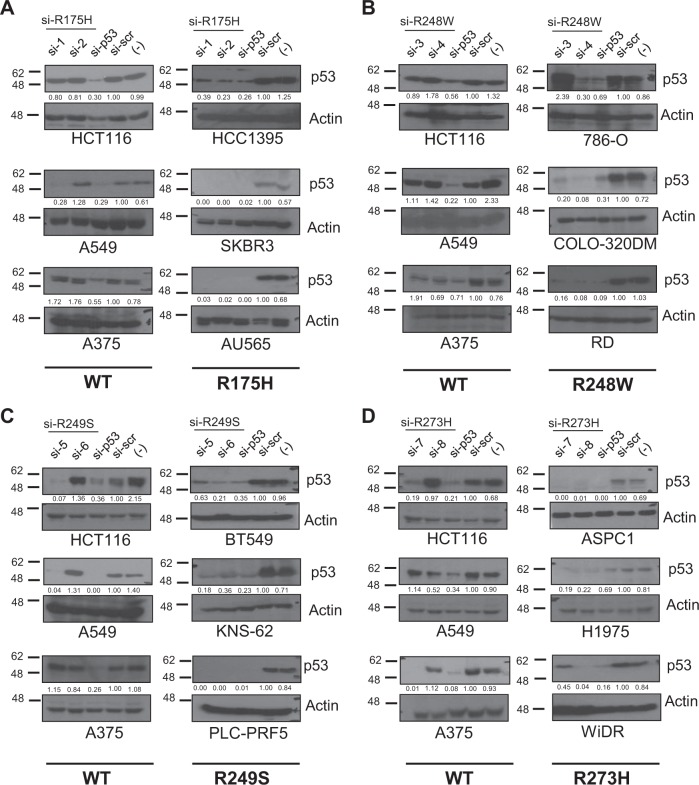
Silencing efficacy of mutant-specific siRNAs on endogenous mutant p53. **a**−**d** siRNAs against R175H (**a**), R248W (**b**), R249S (**c**), and R273H (**d**) were transfected in the three cell lines with WT p53 expression and in three cell lines expressing the indicated p53 mutants, and the silencing efficacy was evaluated by immunoblotting as described above. One representative blot of at least three independent experiments is shown. Mutant p53 status of cell lines is highlighted below the blots and described in Supplementary Table 1

We therefore evaluated the efficacy and specificity of the selected siRNAs on a panel of 17 different cancer cell lines that express either WT or the various p53 mutants (Supplementary Table 1). Similar to the H1299-isogenic cell lines, these cells were transfected with the specific siRNAs or the positive control si-p53 which indiscriminately suppresses the expression of both WT and mutant p53 (Fig. 2a–d). As noted earlier with the H1299-isogenic cell settings, the si-2 was able to specifically downregulate the expression of the R175H mutant in cells expressing this mutant (i.e. HCC1395, SKBR3, and AU565), without having a major impact on the expression of WT p53 in three cell lines (i.e. HCT116, A549, and A375) (Fig. 2a). Similarly, si-4, which is specific for the R248W mutant p53, efficiently inhibited p53 expression in COLO-320DM, 786-O, and RD cells expressing the R248W mutant (Fig. 2b), with no appreciable impact on WT p53 expression in the other cell lines. Similar results were obtained with si-6 which is selective for the R249S mutant in BT549, KNS-62, and PLC-PRF5 cells, and si-8 which is specific for R273H in R273H-expressing ASPC1, H1975, and WiDR cells (Fig. 2c, d). The other siRNAs against the specific mutants, si-1, si-5 and si-7, were also capable of effectively targeting the intended mutants. However, they also displayed significant effects on WT p53 in some cell lines. We therefore further evaluated the specificity of each of the mutant p53-specific siRNAs on various other mutant p53-expressing cells. As shown in Supplementary Figure 1, si-2, si-4, si-6, and si-8 were highly specific and did not affect the expression of the other mutant p53 in all cell lines tested. However, and as noted earlier on the H1299-isogenic cell system, si-1, si-3, si-5, and si-7 had mild or strong impact on other mutants in some cell lines. Collectively, these results highlight that it is possible to generate siRNAs reproducibly that are highly specific and selective for single nucleotide changes but this requires extensive screening to ensure specificity. Based on these analyses, we shortlisted si-2 (for R175H mutant); si-4 (for R248W mutant); si-6 (for R249S mutant); and si-8 (for R273H mutant), for further in-depth characterization.

### Allele-specific knock-down of mutant p53 expression promotes apoptosis and induces p53-target gene expression

**Fig. 3:**
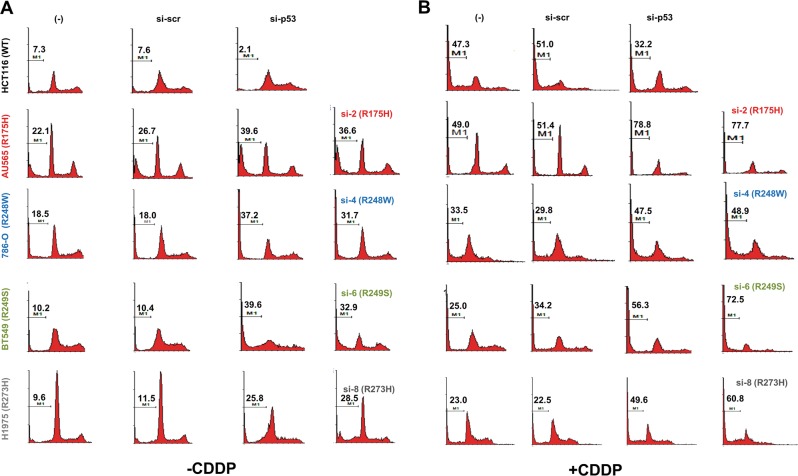
Allele-specific silencing of mutant p53 expression leads to cell death. Flow cytometric analysis of the sub-G1 DNA content (indicative of apoptosis) in cells were quantified 72 h post-transfection of the indicated siRNAs in the indicated cell lines, without (**a**) or with (**b**) CDDP treatment for 24 h. Representative histograms are shown from one experiment out of at least three independent repeats. % sub-G1 cells are indicated in the histogram (M1)

As tumor cells expressing mutant p53 have been shown to be addicted to its presence for survival [26, 27], we first evaluated if the mutation-specific siRNAs will be able to alleviate this phenomenon and induce cell death in the respective mutant-expressing cancer cell lines. Transfection of the specific siRNAs in the respective mutant p53-expressing cell lines universally led to increased apoptosis, as determined by the percentage of sub-G1 population (Fig. 3). While untransfected and scrambled siRNA transfected cells gave basal death, transfection of either the pan-p53 siRNA (si-p53), or the specific mutant p53 siRNAs led to increased cell death in the cell lines expressing the respective mutant p53 (% sub-G1 population in si-scr vs. si-p53 vs. si-mutant p53 → AU565: 26.7 vs. 39.6 vs. 36.6; 786-O: 18.0 vs. 37.2 vs. 31.7; BT549: 10.4 vs. 39.6 vs. 32.9; H1975: 11.5 vs. 25.8 vs. 28.5) (Fig. 3a). Importantly, si-p53 reduced cell death in WT p53-expressing HCT116 cells (si-scr vs. si-p53: 7.6 vs. 2.1), confirming that silencing of mutant p53 expression by a generic p53 siRNA or the mutation-specific siRNA leads to enhanced cell death only in mutant p53-expressing cancer cell lines. Cross-evaluation of the siRNAs on cancer cells expressing other p53 mutants also confirmed their specificity in silencing only the intended mutants, but not on others significantly (Supplementary Figure 2). Concurrent treatment of these cells with the chemotherapeutic agent cisplatin (CDDP) potentiated the cell death induced by the mutation-specific siRNA only in mutant p53-expressing cancer cell lines, but not in WT p53-expressing HCT116 cells (Fig. 3b). Together, these data indicate that cell death induced by silencing mutant p53 further synergizes with cytotoxic drug treatment.

**Fig. 4:**
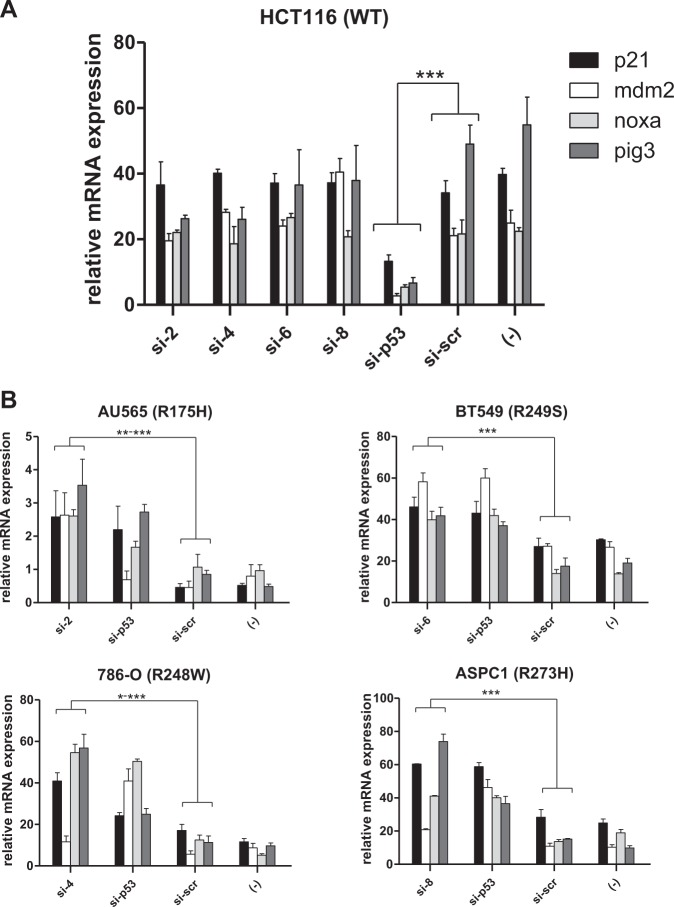
Mutant p53-specific silencing leads to activation of p53 canonical target genes in mutant p53-expressing cells. **a**, **b**. HCT116 cells expressing WT p53 were transiently transfected with siRNAs targeting the four hot-spot p53 mutants or the control scrambled siRNA or p53-specific siRNA. Cells were collected 72 h later for mRNA analysis of the indicated target genes by quantitative real-time PCR (**a**). AU565, 786-O, BT549, and ASPC1 cell lines expressing the indicated p53 mutants were similarly transfected and analyzed (**b**). Relative expression of the target genes is shown. All experiments were normalized to GAPDH and carried out in triplicates. Bar diagrams show the mean ± standard deviation of three independent experiments. * indicates *p* value of <0.05; **<0.005; and ***<0.001, with *n* = 3 samples per group

We and others have previously shown that silencing of mutant p53 in mutant p53-expressing cell lines leads to induction of the expression of canonical p53-target genes, concomitant to the attenuation of the addiction to mutant p53 for survival, in a mechanism involving hypo-acetylation of target gene promoters [27, 28]. We therefore evaluated if this phenomenon also occurs in the context of mutant p53-specific siRNA treatment. To this end, we performed quantitative RT-PCR (qPCR) for several p53 target genes such as *p21, Mdm2, Noxa*, and *Pig3* (Fig. 4). As expected, expression of mRNAs for all the tested p53 target genes was significantly downregulated following p53 silencing in WT p53-expressing HCT116 cells, but were minimally altered by the mutant-specific siRNAs in these cells (Fig. 4a). By contrast, transfection of the mutant-specific siRNAs (i.e. si-2, si-4, si-6, and si-8) or the general p53 siRNA in mutant p53-expressing cells lines led to a significant upregulation of almost all the target genes tested (Fig. 4b). Similar results were obtained using the mutant-specific siRNAs on a different set of cell lines expressing the corresponding mutants (Supplementary Figure 3). Moreover, as noted with the cell viability experiments, concurrent treatment of cells with mutant p53-specific siRNAs with CDDP led to enhanced induction of p53 target genes, highlighting synergy. Furthermore, inhibition of p53 expression in WT p53-expressing cells treated with CDDP led to the expected reduction in target gene expression, indicating the specificity of the effect of the mutant p53 siRNAs on mutant p53-expressing cell lines.

### Inhibition of mutant p53 expression using mutant p53-specific shRNA expression vectors

**Fig. 5:**
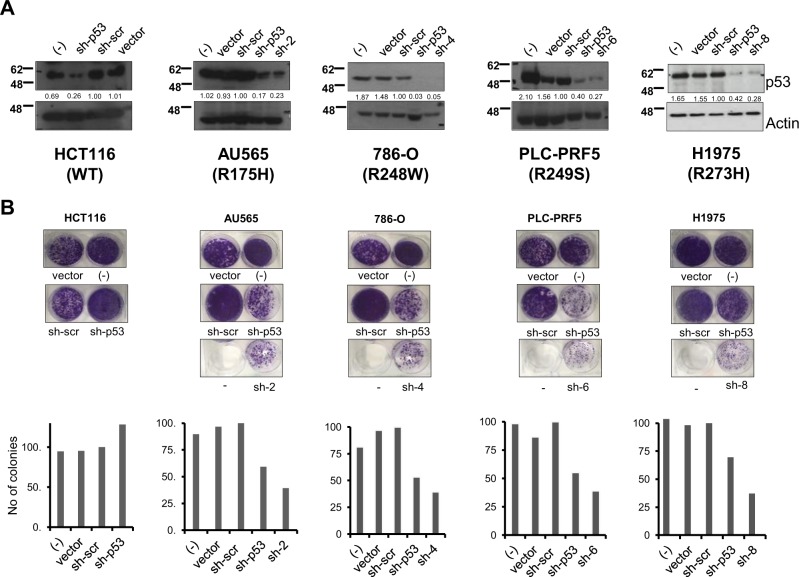
Growth suppressive effect of mutant p53-specific shRNAs. **a**, **b**. The indicated cell lines were transfected with shRNA expressing pan-p53 (sh-p53) shRNA, scrambled control (sh-scr), the respective mutation-specific shRNAs, or empty vector or nothing (−), and harvested 48 h later and analyzed for efficiency of silencing by immunoblotting (**a**). Parallel cultures of cellular colonies were stained with crystal violet solution 2 weeks post shRNA transfection and visualized. Representative images are shown from one experiment, out of at least three independent experiments (**b**), and quantified

To evaluate the long-term effects of the mutant p53-specific silencing, we generated short-hairpin RNAs that express the mutant p53-specific sequences from si-2, si-4, si-6, and si-8 siRNAs, as well as the general p53-specific siRNA and the scrambled siRNA, using the pSuper vector [40]. Initial tests evaluating their efficacy in silencing the expression of the specific mutant p53 were performed in the respective mutant p53-expressing cells lines, after transient transfection of the plasmids. Immunoblot analyses indicated that the mutant p53-specific shRNAs were equally potent in suppressing the expression of the intended mutant p53 in the respective cell lines, unlike the control scrambled shRNA (Fig. 5a). Based on this, we evaluated the effects of suppressing mutant p53 expression on long-term colony growth, which again confirmed that cellular growth was significantly inhibited by silencing the respective mutant p53 (Fig. 5b). Similar results were obtained in short-term apoptosis assays (Supplementary Figure 4A), indicating that shRNA-based mutant p53 silencing is equally effective in promoting cell death of mutant p53-expressing cancer cells.

We also evaluated if the mutation-specific shRNAs are capable of silencing various mutants that occur at the same nucleotide position on *p53*. To test this hypothesis, we utilized the HEC-1A cancer cell line that expresses the R248Q mutation, and transfected the sh-4 which was initially generated against the R248W mutation. As shown in Supplementary Figure 4B-D, sh-4 was capable of silencing the expression of the R248Q p53 mutant, which concomitantly led to increased cell death in short- and long-term assays. These data suggest that mutation-specific si/shRNA against a particular mutated residue is specific for the residue at that position, but does not necessarily discriminate the substituted residues, and hence, can be widely used for the many mutations found at a particular nucleotide position, especially in the case of mutant p53 [2, 11].

### Relief of dominant-negative effect of mutant p53 and enhancement of cell death upon mutant p53 silencing

**Fig. 6:**
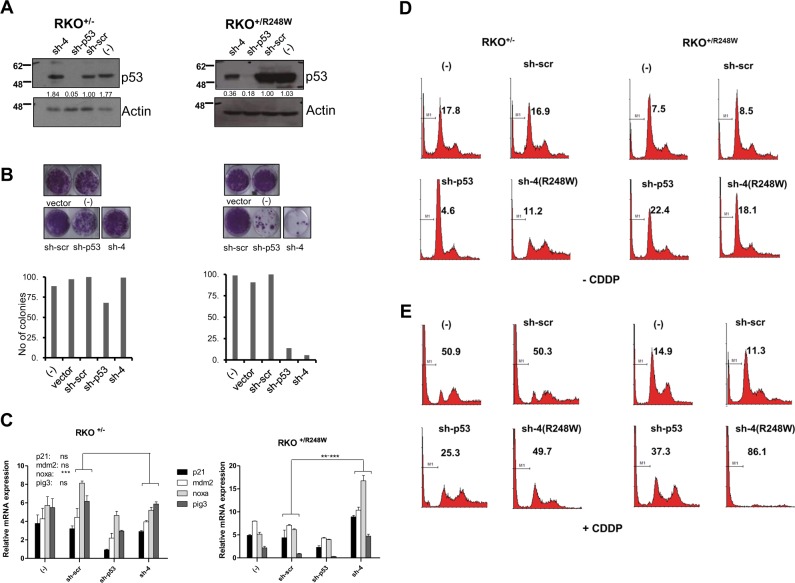
Relief of dominant-negative effects of mutant p53 by mutant p53-specific silencing. **a**−**e**. RKO^+/−^ and RKO^+/248W^ cells were transfected with control, pan-p53 (sh-p53) or R248W-specific shRNAs (sh-4), and analyzed as described above for efficacy of silencing (**a**), colony growth (**b**), and p53 target gene expression (**c**). Cell death was analyzed without (**d**) or with cisplatin (CDDP) treatment (**e**). % sub-G1 cells are indicated on the histograms (as represented by M1). Representative data are shown from three independent experiments and quantified. Bar diagrams show the mean ± standard deviation of the three independent experiments. ** indicates *p* value of <0.005; and ***<0.001, with *n* = 3 samples per group

While expression of mutant p53 alone results in addiction of cancer cells to the mutant protein for survival [26, 27], co-expression of both WT and mutant p53 in the heterozygous state leads to a DN effect of the mutant protein over the WT protein, leading to amelioration of the latter’s functions in target gene activation and apoptosis induction [19, 22, 23]. We have previously shown that reducing the mutant p53 levels in this heterozygous context leads to restoration of WT p53 function, and sensitizes cells to chemotherapeutic agents and irradiation [22]. Hence, we evaluated if the mutant p53-specific shRNAs could be used for reducing mutant p53 levels in mutant heterozygous cells, to improve therapeutic response. To this end, we utilized two sets of isogenic colorectal cells lines (RKO and HCT116), which are heterozygous for p53 (p53^+/−^) or heterozygous for mutant p53 (p53^+/R248W^) [41]. Transfection of sh-4 which is specific for the R248W mutant led to a significant decrease of total p53 in the p53^+/R248W^ cells but not in p53^+/−^ HCT and RKO cells, indicating specificity (Fig. 6a and Supplementary Figure 5A). Concomitant analysis of long-term survival revealed that the sh-4-transfected p53^+/R248W^ cells were more prone to growth inhibition compared to the p53^+/−^ cells (Fig. 6b and Supplementary Figure 5B). Moreover, p53 target gene induction was significantly induced only in the p53^+/R248W^ cells compared to the p53^+/−^ cells when sh-4 was transfected (Fig. 6c and Supplementary Figure 5C), collectively indicating that suppression of mutant p53 relieves the DN effect, and leads to elevated cell death in mutant p53-expressing cells due to the presence of the WT allele.

We also explored the effects of these siRNAs on cell death upon CDDP treatment, which indicated that the presence of mutant p53 reduced cell death (% sub-G1 cells in RKO p53^+/−^ vs. p53^+/R248W^ cells in untransfected and scrambled shRNA transfected: 50.9 and 50.3 vs. 14.9 and 11.3; in HCT cells: 61.1 and 51.2 vs. 32.1 and 28.6), highlighting the DN effects (Fig. 6d, e and Supplementary Figure 5D-E). By contrast, transfection of mutant-specific sh-4 led to a significant increase in cell death particularly in the p53^+/R248W^ cells compared to the p53^+/−^ cells (% sub-G1 cells in RKO p53^+/−^ cells, untransfected vs. sh-4 shRNA: 50.9 vs. 49.7; in RKO p53^+/R248W^ cells: 14.9 vs. 86.1; in HCT p53^+/−^ cells: 61.1 vs. 68.8; in HCT p53^+/R248W^ cells: 32.1 vs. 66.9) (Fig. 6e and Supplementary Figure 5E). These data together demonstrate that silencing mutant p53 specifically without impacting WT p53 expression leads to relief of DN effects and sensitizes mutant-p53-expressing cells to death, which is enhanced by chemotherapeutic drug treatment.

### Therapeutic targeting of mutant p53 retards tumor growth in vivo

**Fig. 7:**
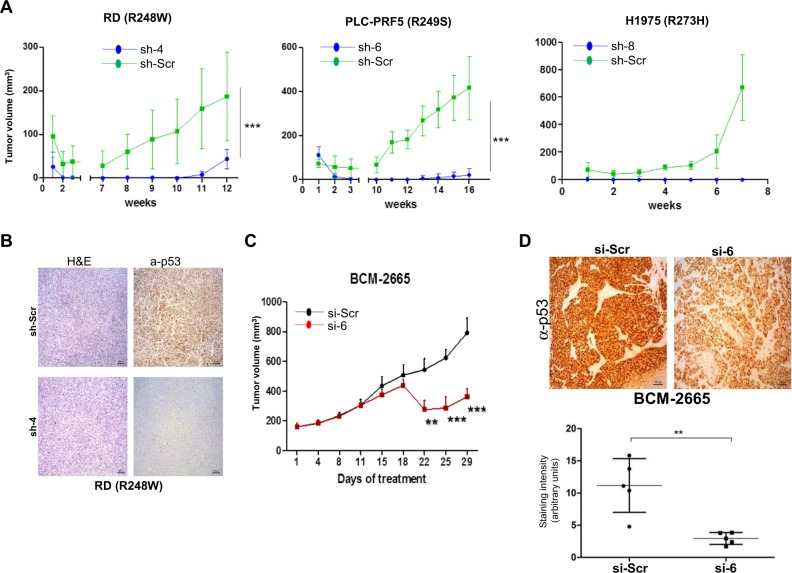
Mutant p53-specific silencing retards tumor growth in vivo. **a**, **b**. RD, PLC-PRF5, and H1975 cell lines were transduced with scrambled or the indicated mutant-p53-specific shRNAs and were collected 3 days later, and cells [RD (4 × 10^6^), PLC-PRF5 (3 × 10^6^) and H1975 (5 × 10^6^)] as a mixture of 75 μl cells in PBS and 75 μl Matrigel were injected into the flanks of SCID mice, and tumor growth was monitored regularly. Sizes of tumors are indicated in the graphs (**a**). Tumors harvested at end point in each case were used for H&E or anti-p53 staining on RD tumors (**b**). Values represent mean + SD. *n* = 4 (per group for RD and H1975 cells) and *n* = 5 (for PLC cells). *** indicates *p* value of <0.001. **c**, **d** Patient-derived triple-negative breast cancer xenografts were generated with 3–5 × 10^6^ cells as a mixture of 50 μl cells in PBS and 50 μl Matrigel and injected orthotopically into 8-week-old C.B-17 SCID mice (*n* = 5 mice per group). Mice were treated with siRNA admixed with liposomes (5 µg/mice), by tail vein injection, twice per week, and monitored regularly (**c**). Tumors harvested at end point in each case were used for anti-p53 staining (**d**). Representative images are shown. Quantitation of p53 staining is shown below. Values represent mean + SD. ** indicates *p* value of <0.01; *** indicates *p* value of <0.001

We finally evaluated if the mutant p53-specific siRNAs would be effective in retarding tumor growth in vivo, by utilizing two models. In the first case, we used the cell-based xenograft model to monitor the growth of cancer cell lines (RD, PLC-PR5 and H1975) expressing the scrambled or the respective mutant-specific shRNAs. Cancer cells which express the various p53 mutants and were transiently infected with viral particles expressing the scrambled shRNA grew to a large volume over time, whereas cells expressing the respective mutant p53-specific shRNAs were markedly retarded in growth in vivo (Fig. 7a). Immunohistochemical analysis for p53 revealed that the mutant-specific shRNA-expressing tumors had significantly reduced *p53*, indicating that the specific shRNAs are effective in silencing the expression of the respective mutant p53 in vivo during tumor growth (Fig. 7b).

In the next model, we examined if the growth of R249S mutant expressing, patient-derived triple-negative breast cancer tumors (PDX) can be influenced by the siRNAs utilized in a therapeutic treatment protocol. In essence, PDX tumors were grown orthotopically, and when they reached 170 mm^3^, mice were treated twice weekly with scrambled siRNA or mutant p53-specific siRNA that was delivered intravenously in nano-liposomes, which have been shown to effectively deliver to tumors [42, 43]. Treatment with si-6 (against R249S) twice weekly led to growth retardation of the tumors when compared to scrambled siRNA-treated mice, which developed to full-blown tumors by 29 days post-treatment (Fig. 7c). Immunohistochemical staining for p53 indicated that the expression of mutant p53 was significantly reduced in the si-6-treated tumors (Fig. 7d), confirming previous results obtained with the xenografts, and highlighting target engagement. Further analysis of multiple organs at sacrifice from siRNA-treated mice did not show any abnormalities, excluding any side effects due to this treatment regimen (data not shown). Taken together, these data establish that mutant p53-specific siRNAs can be used therapeutically to retard tumor growth in vivo.

## Discussion

The results presented here demonstrate that siRNAs that are highly specific and capable of distinguishing one nucleotide change can indeed be regularly generated, and highlight their utility in targeting four p53 hot-spot mutants. These four p53 mutants account for about 20% of all p53 mutations found in cancers [2, 11], and targeting them represents the possibility of targeting about 10% of all cancers. Targeting mutant p53 resulted in improved chemo-sensitivity, as it had minimal or no effects on the WT p53 protein in the heterozygous cells, allowing the latter to function to induce cell death. Furthermore, abrogation of mutant p53 expression in cancer cells expressing only mutant p53, as often seen in later stages of cancers where the WT p53 allele is lost due to loss-of-heterozygosity [44, 45], resulted in retardation of tumor growth in vivo even when used as a mono-therapy. These data highlight the huge therapeutic potential that could be harnessed with these MupSis, whose effects could likely be further enhanced in combination with other chemotherapeutic agents or radiotherapy. Hence, these data provide the impetus to target mutant p53 directly for clinical benefit, which could be translated to the clinical settings soon.

The era of precision medicine has promoted the development of many drugs that are specific for the highly active mutant forms of proteins, and has resulted in huge clinical benefits, as in the case of leukemia with Bcr-Abl translocations (treated with Imatinib); and lung cancers with EGFR mutations (gefitinib), amongst others [4, 46]. While spectacular results have initially been achieved, two major issues with specificity remain. Firstly, the drugs generated against a particular protein (often kinase) almost always have an impact on other cellular targets, as very recently shown by the analyses of 37 kinase inhibitors [8]. Moreover, many of these drugs, though very effective on the mutant and active forms of the proteins, also have significant impact on the WT counterparts, as has been shown for c-Kit [7]. Therefore, though effective, the impact of these inhibitors on the WT form or other closely related targets will unabatedly lead to unwanted side effects, reducing the promise of these reagents.

In this respect, we have recently developed monoclonal antibodies against three of the above described four hot-spot p53 mutations [47], as part of our program to develop reagents that are capable of recognizing single amino-acid or nucleotide changes associated with pathological conditions. As a proof-of-principal to demonstrate their utility, we have shown that the p53 mutation-specific antibodies are highly specific and are capable of recognizing the intended mutant forms without any cross-reactivity to the WT form or other closely related mutants [47]. We have shown that these antibodies are effective in recognizing samples with the specific mutations, and hence, could be used for pathological diagnosis. These antibodies could therefore be used as companion diagnostics together with the afore-described MupSis, which can be used for therapeutic targeting. Efforts are currently ongoing to generate similar reagents to an array of the most common genetic alterations and SNPs found in cancers and other diseases.

siRNAs have been generated successfully to silence gene expression and has been extensively used in research, and also been translated to the clinical setting [48, 49]. Most of these siRNAs target the whole gene (protein), without cross-reactivity to other related genes. However, only few examples exist for the generation of siRNAs that are capable of discerning single nucleotide changes found in the disease states. The ones that have been generated with some specificity for single nucleotides include those against mutant keratin, p63, dynamin, as well as R248W mutant p53 [35–39, 50]. They have been shown to be relatively specific in reporter assays and in overexpression systems, though some level of cross-reactivity with the WT protein is often noted. Moreover, many of them have not been tested in a large number of cell lines to establish their specificity unequivocally. These factors highlight the enormous challenges in obtaining siRNAs that show specificity at the nucleotide level, and which can be used on critical genes that affect a multitude of process in normal physiology, like p53. Our work has revealed that a huge library of siRNAs has to be tested prior to obtaining highly specific ones, especially since the effects of the addition or subtraction of a few nucleotides in the siRNA sequences can make a huge difference. This is highlighted by our results using the siRNAs against R248W. Of these, si-3, which is similar to that published earlier [35], has some effects of WT p53 or R175H mutant expressing cells, though being capable of fully reducing the expression of R248W (Figs. 1 and 2; and Supplementary Figure 1). By contrast, si-4 is extremely specific only for the intended R248 mutant. This demonstrates that very subtle changes in the sequences of the siRNA significantly affects the specificity and leads to marked differences in selectivity, and highlights that one cannot intuitively predict the effects of the various sequences. Though it is relatively possible to obtain siRNAs that appear to be nucleotide specific (such as si-3), especially when assayed against one or two cell lines or using transfection systems, analyses against a large panel of cellular systems is essential to ensure that they are really specific. This becomes very crucial when these are to be used in the clinical setting. Hence, the set of siRNA sequences presented here represents a unique set of siRNAs that are capable of specifically targeting almost 20% of all cancers with mutations in *p53*, supporting the notion that with sufficient screening, nucleotide-specific siRNAs can be generated and evaluated in clinical trials, as has been demonstrated with the mutant keratin-specific siRNAs to treat the skin disorder Pachyonychia Congenita [51].

We have chosen mutant p53 to demonstrate the ability to generate nucleotide-specific siRNAs, as it is the most mutated gene across all cancers. Importantly, not all p53 mutants behave similarly [23], and thus, targeting mutant p53 requires selective agents to target each of them individually. Moreover, targeting mutant p53 represents a huge untapped route to retard tumor cell growth and metastasis, and to improve sensitivity to general cytotoxic agents, and would therefore find applicability against most cancer types. As highlighted earlier, mutant p53 can exist either with the WT allele in the earlier stages of tumorigenesis, or by itself after the loss of the WT allele due to LOH in later stages. In the earlier stages, mutant p53 inhibits the WT protein through the DN effect, and at the later stages, the mutant provides a survival advantage independent of the WT allele. Our data demonstrate that the mutant p53-specific siRNAs are capable of relieving both the DN effect as well as the addiction of cancer cells to mutant p53, and could thus be used widely as long as the mutation is present in the tumors. Similarly, targeting other driver oncogenes with specific siRNAs in conjunction with mutant p53 will likely enhance the therapeutic effects, and one could envisage a cocktail of siRNAs against the major genetic alterations in each cancer type would likely be clinically very beneficial.

In conclusion, we have demonstrated the generation and characterization of siRNAs that are extremely specific for the various p53 mutants that are highly represented in human cancers. These data could be translated directly for clinical evaluation with the appropriate delivery mechanisms, some of which are in clinical trials [52]. Furthermore, this work also provides the necessary impetus to generate many similar reagents to target only the mutant forms of proteins altered in disease sates, absolutely minimizing cross-reactivity, and thus, reducing side effects associated with many of today’s cancer drugs.

## Materials and methods

### siRNA design

A large library of siRNAs was designed to target p53 hot-spot mutations (R175H, R248W, R249S, and R273H), out of which eight were shortlisted for the four mutants for further characterization (si-1−8). One siRNA against all *p53* alleles generated in our screen was used as a positive control for pan-p53 targeting. Control scrambled siRNA had no bio-informatically predicted sequence target in the human genome and was used as a negative control.

### Design of shRNA template oligonucleotides and construction of plasmid

shRNA target sequences were designed to be homologous to the siRNA sequences afore-described. The pRetro-Super vector contains a human H1 polymerase-III (pol-III) promoter for shRNA expression [40, 53]. Each shRNA insert was designed as a synthetic duplex with overhanging ends identical to those created by restriction enzyme (RE) digestion (*Bam*HI at the 5′ and *Hin*dIII at the 3′). The coding region for each hairpin is nested within a single oligonucleotide (upper oligo: 5′-AAGCTTTN [19–29] (sense sequence)TTCAAGAGAN [19–29] (antisense sequence)TTTTTTA-3′) and its complementary equivalent (lower oligo: 5′-AGCTTAAAAAN [19–29] (sense sequence)TCTCTTGAAN [19–29] (antisense sequence)GGG-3′). These ranged in size from 60 to 100 bases (for hairpins with 19–29 bp stems). Each duplex contained a transcription initiation base, the shRNA encoding region (sense stem, loop sequence and antisense stem), a termination spacer and a pol-III termination signal consisting of a run of at least 4 “T”s. The transcription initiation base was an “A” or “G” (required for efficient pol-III transcription initiation) and was only included if the first base of the hairpin stem was not a purine. The termination spacer was any base but “T” and was included only if the last base of the antisense stem was “T” so as to prevent premature termination via an early run of “T”s. Oligos were ordered at the minimal synthesis and purification scales (0.05 μM and desalt, Sigma-Aldrich). Each oligo was re-suspended in water at a 100 μM concentration and 10 μl from each was added to 20 μl of 2× annealing buffer (200 mM potassium acetate, 60 mM HEPES_KOH pH 7.4, 4 mM Mg-acetate), heated to 95 °C for 10 min, slowly equilibrated to room temperature and diluted 1:1000 fold for ligation. The insert and vector were ligated, and transformed into TOP10 or DH5α competent *cells*. Clones with the shRNA insert were selected and purified before transfection.

## Supplementary information

Supplemental Text

Supplemental Figure and Table
