# Supplementary material for: Cancer therapeutic targeting using mutant–p53-specific siRNAs
Source: Oncogene. 2019 Jan 14;38(18):3415–27. doi: 10.1038/s41388-018-0652-y (PMC6756012; doi:10.1038/s41388-018-0652-y)
Supplement: Supplementary file 1 — Supplemental Text [file 41388_2018_652_MOESM1_ESM.docx]

**Supplementary Information**

**Supplementary Materials and Methods**

***Cell culture***

Cell lines were obtained from ATCC and JCRB and were cultured under standard conditions (37°C, 5% CO2) with the following media: DMEM with 4.5 g/L glucose and 10% FBS (Hyclone) for H1299, RKO, HCT116, A549, A375, SKBR3, RD, PLC-PRF-5, KNS-62 and HEC1A cell lines; RPMI-1640 and 10% FBS (Hyclone) for AU565, HCC1395, COLO-320DM, 786-O, ASPC-1 and WiDR and H1975; RPMI-1640 with 0.023 IU/ml insulin and 10% FBS (Hyclone) for BT-549; RKO p53^+/-^ and ^+/R248W^ and HCT p53^+/-^ and ^+/R248W^ cell lines are a kind gift from Dr Bert Vogelstein (41).

***Transfection of p53 siRNA/shRNA, and RNA and protein analyses***

2.5 x 10^5^ cells per well were seeded in a 6-well plate 24h prior to transfection. Cells were transfected with 80nM siRNA or 1μg of the pRetroSuper-shRNAs using LipofectamineTM 2000 reagent (Invitrogen) as per the manufacturer’s manual. Each transfection was performed in triplicates and the cells were harvested with 1mL of TRIzol reagent (Invitrogen) 72h after transfection.

Total RNA isolation was performed using Invitrogen’s standard protocol, and cDNA was prepared using Superscript II reverse transcription (Invitrogen). Quantitative real time (RT)-PCR analysis was performed on the following p53 target genes: *p21, pig3, mdm2, noxa* and gapdh, as described (54).

Cell extracts were prepared in lysis buffer (0.7% NP40; Tris.HCl, pH 7.4; 70 mM EDTA; 200 nM NaCl on ice for 10 min). After protein quantification, 30-50μg of lysate was loaded on SDS–polycrylamide gel (12%) electrophoresis (SDS–PAGE), and the resolved proteins were transferred electrophoretically to polyvinylidene fluoride (PVDF) membranes (Invitrogen, Breda, The Netherlands). The detection of the protein was done with ECL (GE Healthcare, Waukesha, WI, USA). p53 was detected with a mouse anti-p53 monoclonal antibody (DO-1 from Santa Cruz Biotechnology, #SC126) and actin was detected with a rabbit anti-actin antibody (Sigma, #82066). Paralleled gels were run with equal amounts of lysates and probed with the various antibodies separately, in cases where background from the first antibody was high. Quantification of western blots was done using the ImageJ software by lane plotting and peak labelling (signal intensity quantification). For each sample, the ratio of p53 to Actin band intensity was calculated and normalized to the ratio of si-scr/sh-scr control. Values represent normalized fold change.

***Cell death assays***

Cells were transfected with 80nM siRNA and harvested 72h post-transfection, including floating cells in the medium. Cells were washed 2X in PBS and were fixed in 70% ethanol overnight and subsequently treated with RNase for 20 minutes before addition of 5μg/ml propidium iodide (PI) and flow cytometric analysis was performed (BD Biosciences FACScalibur), to measure apoptosis (sub-G1 DNA content).

***Colony formation assay***

The indicated cell lines were transfected with the indicated shRNA plasmids containing oligonucleotide sequences for silencing the various p53 mutants and were selected for 2 weeks on 15μg/ml of Blasticidine (Sigma, USA). Colonies were stained with crystal violet solution (Merck), as described.

***Generation of shRNA expressing cell lines for in vivo tumor growth analysis***

Viruses for p53 mutant-specific shRNAs were generated using pCL-Ampho amphotropic virus packaging plasmid in HEK293T cells. Briefly, retroviruses were prepared by transfection of HEK293T cells with the 1.5μg of the appropriate shRNA and 1μg of the packaging plasmid using lipofectamine 2000™. Retroviral supernatants were harvested 24h after transfection, filtered through 0.45μM syringe filter, aliquoted and frozen. 3.5ml of retroviral supernatant was used to transduce 5 x 10^5^ cells in a 10cm dish in the presence of 10mg/ml of polybrene (Sigma) in duplicates in 6cm dish. A second transduction was performed the following day. Cells were selected using 10μg/ml of blasticidine for 48h after the second transduction, and harvested for in vivo xenograft studies. Parallel cultures were used for immunoblots analysis to assess the efficiency of p53 knockdown.

Cell lines expressing the respective shRNA were harvested, and mixed with Matrigel in a ratio of 1:1 on ice (Corning®Matrigel® basement membrane matrix, Sigma), and 150ul total volume subcutaneously injected in the right flank of female C.B-17 SCID mice (6-8 weeks of age), and cells transduced with the scrambled shRNA were injected on the left flanks of each mice. Tumor volume was assessed with a caliper twice per week and values were taken down as soon as tumors became palpable. Calculation of tumor volume was done according to V=1/2*(longer side*shorter side^2). Values are plotted as means with standard deviation. Statistical significance between growth curves was calculated with PRISM software (GraphPad Prism Software Inc., San Diego, CA) using unpaired (two-tailed) t-test. Four to five mice per group were used for each treatment. When mice were sacrificed, tumor tissues were excised and fixed in 10% formalin over-night, dehydrated and embedded in paraffin and 5μm sections were prepared. Anti-p53 staining was done using p53 1C12 Mouse monoclonal antibody (Cell Signaling Technology, #2524) with a concentration of 1:1500. Staining signal was developed using Dako REAL™ EnVision™ Detection System, Peroxidase/DAB+, Rabbit/Mouse (#5007). All animal experiments were conducted as approved by the SingHealth Institutional Animal Care and Ethics Committee.

Histologically confirmed patient-derived xenografts (PDX) were used to confirm the data. PDXs were implanted into the right mammary fat pad of 4-5-week-old female severe combined immunodeficiency beige mice (Harlan Laboratories, Madison, WI, USA). Once tumor volume reached 150 to 200 mm3, mice (n=5 per group) were randomly assigned to receive scrambled or mutant p53 R249S-specific siRNA (5μg/mice) admixed in nano- liposomes by tail vail injection, twice a week, as described (42,43). Tumor volumes were measured twice weekly. All animal experiments were conducted as approved by the Houston Methodist Hospital’s Animal Care and Ethics Committee.

***Statistical analysis***

Statistical analysis was done with GraphPad PRISM software, using unpaired, two-tailed t-test. * indicates ***p*** value of <0.05; **<0.005; and ***<0.001.

**Supplementary Figure Legends**

***Supplementary Figure 1. Silencing efficacy of mutant-specific siRNAs on endogenous mutant p53 expression (Related to Figure 2)***

siRNAs against R175H (si-1 & 2), R248W (si-3 & 4), R249S (si-5 & 6) and R273H (si-7 & 8), were transfected in the various cell lines expressing the indicated p53 mutants, and the silencing efficacy was evaluated by immunoblotting as described. One representative blot of at least two independent experiments is shown. Mutation p53 status of cell lines is highlighted below the blots and described in Supplementary Table 1. For each sample, the ratio of p53 to Actin band intensity was calculated and normalized to the ratio of si-scr control. Values represent normalized fold change.

***Supplementary Figure 2. Evaluation of effects of mutant-specific siRNAs on cell death in cell lines expressing various mutant p53*** ***(Related to Figure 3)***

Flow cytometric analysis of the sub-G1 DNA content (indicative of apoptosis) in cells were quantified 72h post-transfection of the indicated siRNAs (as described) in the indicated cell lines. Representative histograms are shown. % sub-G1 cells are indicted in the histogram (M1 for the HCC1395 cells and M2 for all the other cell lines).

***Supplementary Figure 3. Depletion of mutant allele expression leads to activation of p53 transcriptional targets (Related to Figure 4)***

qRT-PCR for p53 target genes such as *p21, Mdm2* and *Noxa* was performed on the indicated cells lines that were transfected with the various siRNA, and treated without or with cisplatin (CDDP), as described. All experiments were normalized to GAPDH and carried out in triplicates, and relative expression of the target genes is shown. Bar diagrams show the mean ± standard deviation of three independent experiments. * indicates ***p*** value of <0.05; **<0.005; and ***<0.001, with n=3 samples per group.

***Supplementary Figure 4. Allele-specific mutant p53-specific shRNAs induce cell death and are effective on various mutant nucleotides at the same residues (Related to Figure 5)***

**A.** Flow cytometric analysis of the sub-G1 DNA content (indicative of apoptosis) was performed 72h post-transfection in the various cell lines which were transfected with the indicated shRNAs. Representative histograms show the mean ± standard deviation of three independent experiments.

***B-D.*** HEC1A cells expressing the R248Q mutant p53 were transfected with the indicated shRNAs and analyzed for mutant p53 expression (B), colony growth (C) and apoptosis in the absence or presence of CDDP treatment (D). Representative results from one of three independent experiments are shown. Bar diagrams show the mean ± standard deviation of three independent experiments. sh-4 is the R248W specific siRNA.

***Supplementary Figure 5. Relief of dominant-negative effects of mutant p53 by mutant p53-specific silencing (Related to Figure 6)***

**A-E.** HCT116^+/-^ and HCT116^+/R248W^ cells were transfected with control, pan-p53 (sh-p53) or R248W-specific shRNAs (sh-4), and analyzed as described for efficacy of silencing (A), colony growth (B), and p53 target gene expression (C). Cell death was analyzed without (D) or with cisplatin (CDDP) treatment (E). % sub-G1 cells are indicated on the histograms (as represented by M1). Representative data are shown from three independent experiments. Bar diagrams show the mean ± standard deviation of the three independent experiments. * indicates ***p*** value of <0.05; **<0.005; and ***<0.001, with n=3 samples per group.
