# Supplementary material for: Cancer therapeutic targeting using mutant–p53-specific siRNAs
Source: Oncogene. 2019 Jan 14;38(18):3415–27. doi: 10.1038/s41388-018-0652-y (PMC6756012; doi:10.1038/s41388-018-0652-y)
Supplement: Supplementary file 2 — Supplemental Figure and Table [file 41388_2018_652_MOESM2_ESM.pdf]

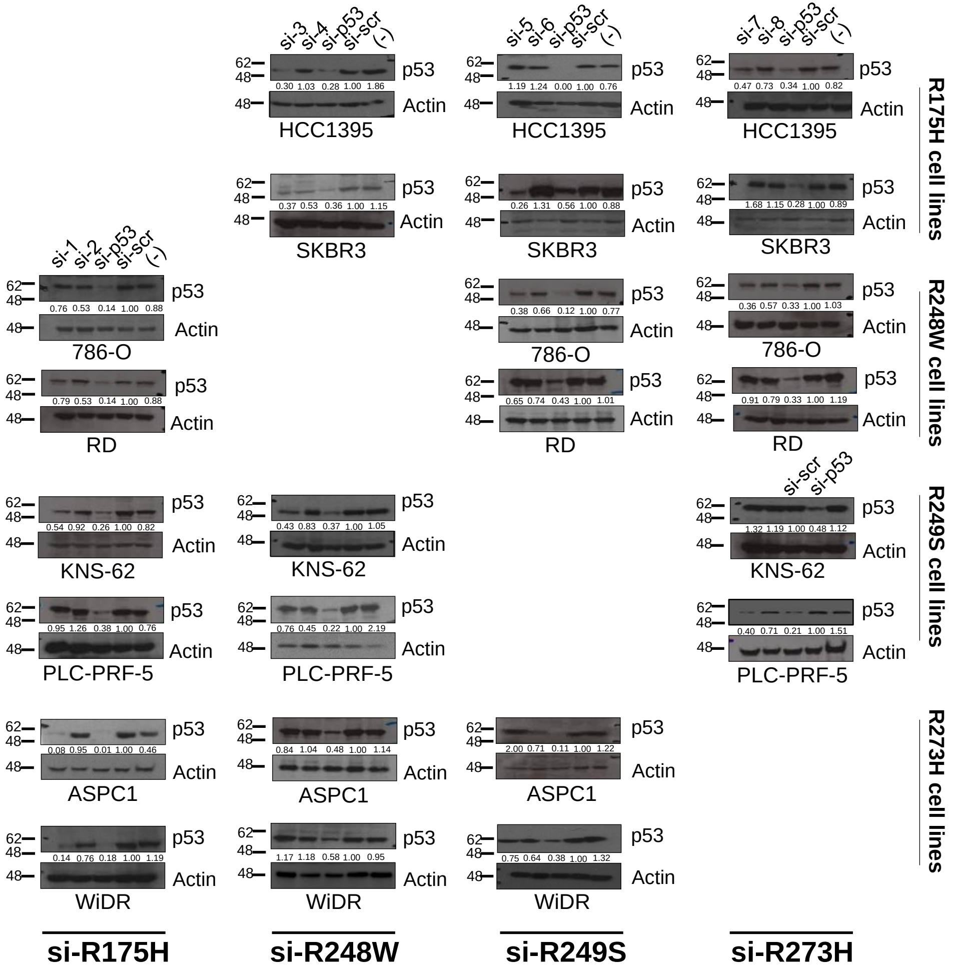

Supplementary Figure 1 Ubby et al.

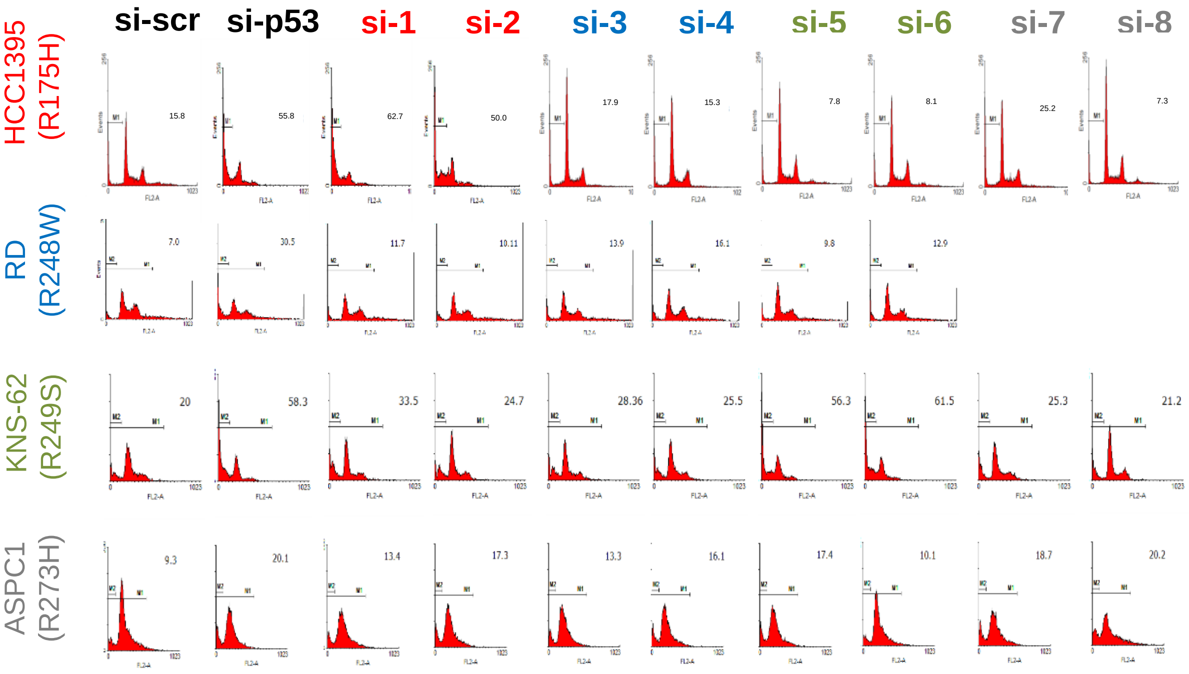

Supplementary Figure 2 Ubby et al.

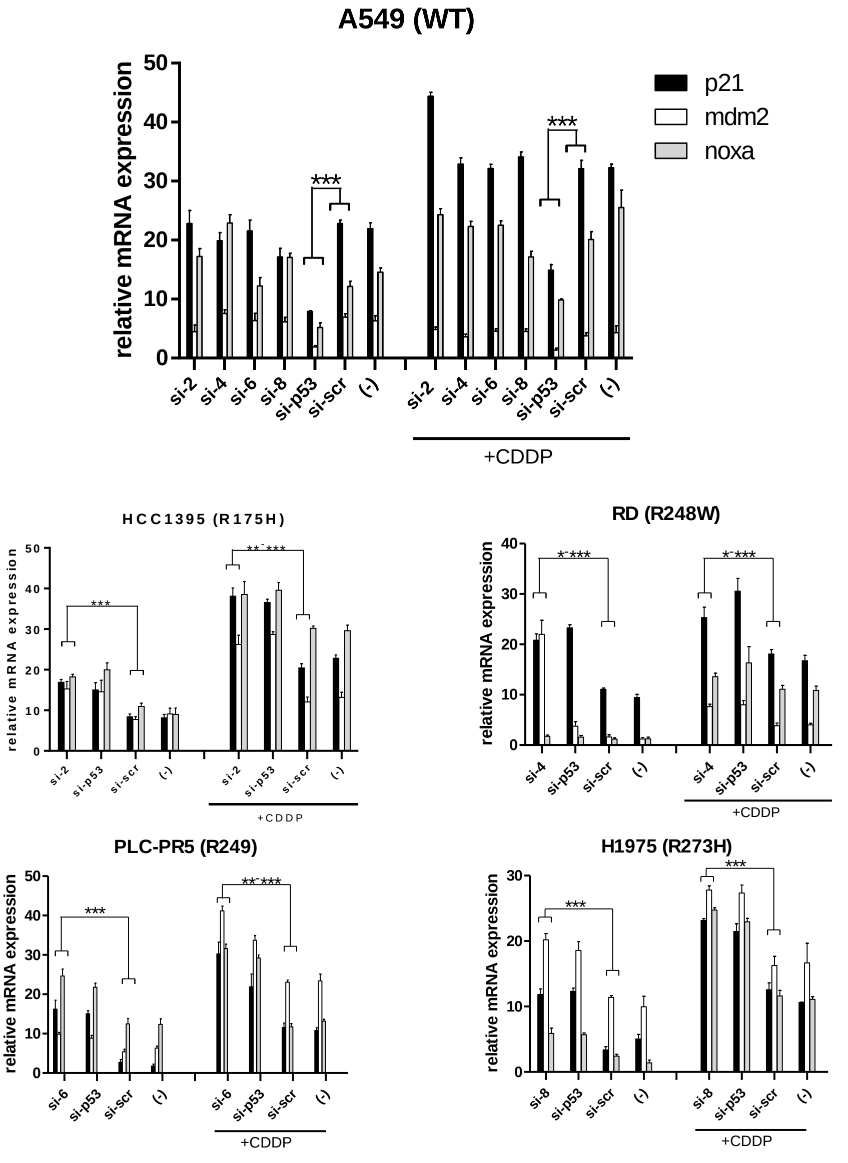

Supplementary Figure 3 Ubby et al.

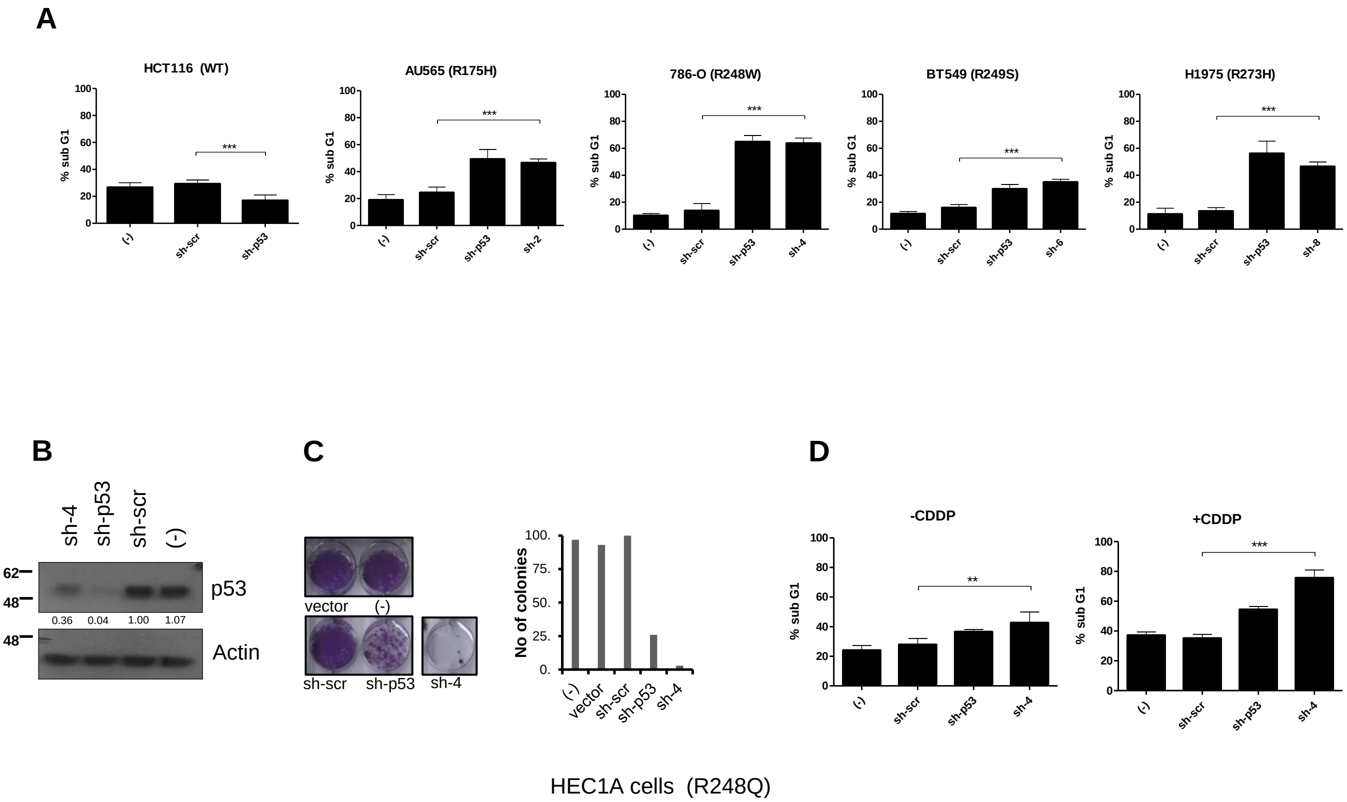

Supplementary Figure 4 Ubby et al.

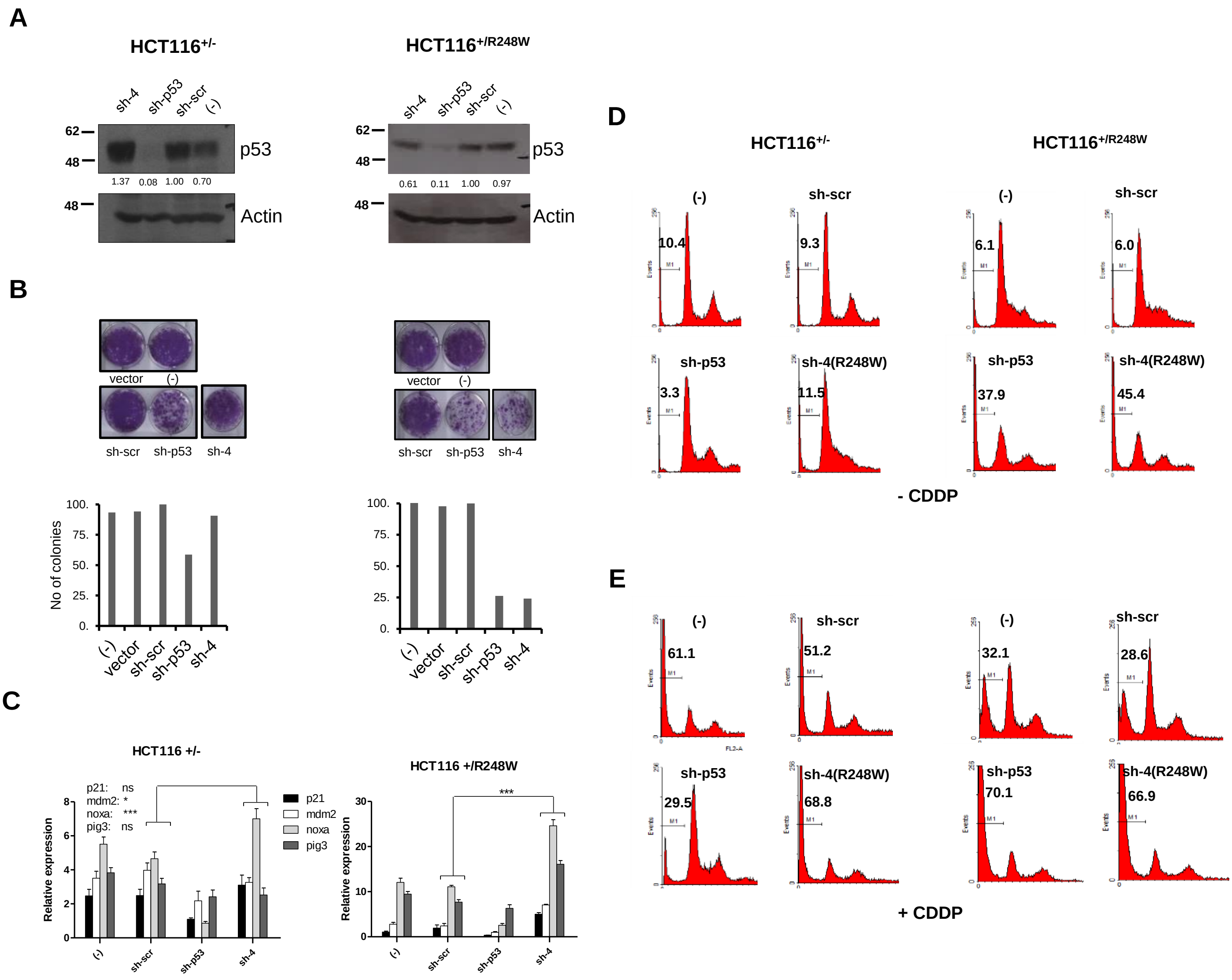

Supplementary Figure 5 Ubby et al.

Supplementary Table 1: Human tumor cell lines used in the study

Cell lines used in study	p53 status	Origin of cell line
A549	Wild-type	Lung carcinoma
A375	Wild-type	Malignant melanoma
HCT116	Wild-type	Colon carcinoma
RKO	Wild-type	Colon carcinoma
AU565	R175H	Breast carcinoma
SKBR3	R175H	Breast carcinoma
HCC1395	R175H	Breast carcinoma
HEC-1A	R248Q	Endometrial adenocarcinoma
RD	R248W	Rhabdomyosarcoma
786-O	R248W	Kidney adenocarcinoma
COLO-320DM	R248W	Colon adenocarcinoma
PLC-PRF/5	R249S	Hepetocellular carcinoma
KNS-62	R249S	Bronchial squamous cancer
BT549	R249S	Breast carcinoma
ASPC1	R273H	Pancreatic adenocarcinoma
WiDR	R273H	Colon adenocarcinoma
H1975	R273H	Lung carcinoma
